# Supplementary material for: Olive Oil-Based Oleogel as Fat Replacer in a Sponge Cake: A Comparative Study and Optimization
Source: Foods. 2022 Aug 31;11(17):2643. doi: 10.3390/foods11172643 (PMC9455797; doi:10.3390/foods11172643)
Supplement: Supplementary file 1 [file foods-11-02643-s001.zip › foods-1865889-supplementary.pdf]

Olive Oil-Based Oleogel as Fat Replacer in a Sponge Cake: A Comparative Study and Optimization

Francesca Malvano [1], Mariachiara Laudisio [1], Donatella Albanese [1,*], Matteo d'Amore [2] and Francesco Marra [1]

[1] Department of Industrial Engineering, University of Salerno, 84084 Fisciano, SA, Italy
[2] Department of Pharmacy, University of Salerno, 84084 Fisciano, SA, Italy
* Correspondence: dalbanese@unisa.it

Supplementary Data

Equation **(S1)**. $Hardness = 2.11 * \left(\frac{(x_1-0.65)}{0.35}\right) - 11.28 * \left(\frac{x_2}{0.35}\right) + 3.14 * \left(\frac{x_3}{0.35}\right) + \left(\frac{(x_1-0.65)}{0.35}\right) * \left(\left(\frac{x_2}{0.35}\right) * 17.13\right) + \left(\frac{x_1-0.65}{0.35}\right) * \left(\left(\frac{x_3}{0.35}\right) * 0.16\right) + \left(\frac{x_2}{0.35}\right) * \left(\left(\frac{x_3}{0.35}\right) * 23.61\right) + \left(\frac{(x_1-0.65)}{0.35}\right) * \left(\left(\frac{x_2}{0.35}\right) * \left(\left(\frac{x_3}{0.35}\right) * -23.61\right)\right)$

Equation **(S2)**. $a_w = 0.84 * \left(\frac{(x_1-0.65)}{0.35}\right) + 0.75 * \left(\frac{x_2}{0.35}\right) + 0.81 * \left(\frac{x_3}{0.35}\right) + \left(\frac{(x_1-0.65)}{0.35}\right) * \left(\left(\frac{x_2}{0.35}\right) * 0.12\right) + \left(\frac{x_1-0.65}{0.35}\right) * \left(\left(\frac{x_3}{0.35}\right) * -0.11\right) + \left(\frac{x_2}{0.35}\right) * \left(\left(\frac{x_3}{0.35}\right) * 0.08\right) + \left(\frac{(x_1-0.65)}{0.35}\right) * \left(\left(\frac{x_2}{0.35}\right) * \left(\left(\frac{x_3}{0.35}\right) * 0.12\right)\right)$

Equation **(S3)**. $Porosity = 74.67 * \left(\frac{(x_1-0.65)}{0.35}\right) - 132.57 * \left(\frac{x_2}{0.35}\right) + 75.74 * \left(\frac{x_3}{0.35}\right) + \left(\frac{(x_1-0.65)}{0.35}\right) * \left(\left(\frac{x_2}{0.35}\right) * 316.28\right) + \left(\frac{x_1-0.65}{0.35}\right) * \left(\left(\frac{x_3}{0.35}\right) * 14.57\right) + \left(\frac{x_2}{0.35}\right) * \left(\left(\frac{x_3}{0.35}\right) * 315.63\right) + \left(\frac{(x_1-0.65)}{0.35}\right) * \left(\left(\frac{x_2}{0.35}\right) * \left(\left(\frac{x_3}{0.35}\right) * 17.48\right)\right)$

Equation **(S4)**. $Moistness = 3.38 * \left(\frac{(x_1-0.65)}{0.35}\right) + 4.66 * \left(\frac{x_2}{0.35}\right) + 2.32 * \left(\frac{x_3}{0.35}\right) + \left(\frac{(x_1-0.65)}{0.35}\right) * \left(\left(\frac{x_2}{0.35}\right) * -2.92\right) + \left(\frac{x_1-0.65}{0.35}\right) * \left(\left(\frac{x_3}{0.35}\right) * 0.48\right) + \left(\frac{x_2}{0.35}\right) * \left(\left(\frac{x_3}{0.35}\right) * -4.58\right) + \left(\frac{(x_1-0.65)}{0.35}\right) * \left(\left(\frac{x_2}{0.35}\right) * \left(\left(\frac{x_3}{0.35}\right) * -4.77\right)\right)$
